# First Molecular Identification and Prevalence of *Sarcocystis* spp. in Sheep Intended for Human Consumption in Shanxi Province, China

**DOI:** 10.3390/vetsci11100504

**Published:** 2024-10-14

**Authors:** Yu Kang, Xin-Sheng Lu, Yuan-Hui He, Chen Wang, Ze-Xuan Wu, Lu Wang, Xiao-Jing Wu, Jun-Jie Hu, Xing-Quan Zhu

**Affiliations:** 1 Laboratory of Parasitic Diseases, College of Veterinary Medicine, Shanxi Agricultural University, Jinzhong 030801, China; yukang2024@126.com (Y.K.); luxinsheng2024@126.com (X.-S.L.); sxhyh2024@163.com (Y.-H.H.); wangchen509260@163.com (C.W.); wuzexuan0602@163.com (Z.-X.W.); llyhtw1105@163.com (L.W.); wuxiaojing2017@163.com (X.-J.W.); 2 School of Ecology and Environmental Sciences, Yunnan University, Kunming 650091, China

**Keywords:** *Sarcocystis* spp., prevalence, sheep, Shanxi province

## Abstract

**Simple Summary:**

The infection of animals and humans with *Sarcocystis* spp. causes public health problems and significant economic losses to the livestock industry. To date, more than 200 species in the genus *Sarcocystis* have been reported. *Sarcocystis* spp. can cause sarcocystosis, which leads to emaciation, abortion, and even death in sheep. However, to date, *Sarcocystis* spp. infection has not been reported in sheep (*Ovis aries*) in Shanxi Province. In the present study, 582 mutton samples collected from sheep in ten representative counties in this province were investigated for *Sarcocystis* spp. by PCR amplification of the mitochondrial cytochrome c oxidase subunit I (*cox*1) gene. The results showed that the prevalence of *Sarcocystis* spp. in sheep in Shanxi Province was 33.85% (197/582). Of them, 196 *cox*1 sequences showed a nucleotide similarity of 98.56–99.81% with those of *S. tenella*, and the remaining one *cox*1 sequence showed a nucleotide similarity of 99.71% with that of *S. arieticanis*. The present study reported the occurrence and prevalence of *Sarcocystis* spp. for the first time in sheep in Shanxi Province, China, which has important implications for the control and prevention of *Sarcocystis* infection in this province and elsewhere.

**Abstract:**

*Sarcocystis* species are intracellular coccidian protozoans that can infect a range of animals and humans and cause public health problems and economically significant losses. Sarcocystosis in sheep (*Ovis aries*) can cause abortion, neurological symptoms, and even death and results in significant economic losses to the livestock industry. To date, however, it is yet unknown whether sheep in Shanxi Province, north China, are infected with *Sarcocystis* spp. The purpose of this study was to investigate the prevalence of *Sarcocystis* spp. in sheep in Shanxi Province. Thus, 582 muscle samples of sheep were purchased from farmers’ markets from ten representative counties in Shanxi Province, north China, and examined for the presence and prevalence of *Sarcocystis* spp. by PCR amplification of the mitochondrial cytochrome c oxidase subunit I (*cox*1) gene. Of the examined 582 mutton samples, 197 samples (33.85%) were *Sarcocystis*-positive and were sequenced. Of the obtained 197 *cox*1 sequences, 196 sequences showed nucleotide similarity of 98.56–99.81% with those of *S. tenella*, and the remaining one *cox*1 sequence showed nucleotide similarity of 99.71% with that of *S. arieticanis*. Two representative *cox*1 sequences of *S. tenella* (accession nos. PQ189447 and PQ189448) have 99.52% and 99.61% identity with *S. tenalla* (KC209725) and *S. tenalla* (MK419984), respectively. The sequence of *S. arieticanis* (accession no. PQ165949) obtained in this study has 99.71% identity with *S. arieticanis* (MK419975). This present study documents the occurrence and prevalence of *Sarcocystis* spp. in sheep in Shanxi Province for the first time, which enriches the data on the distribution of *Sarcocystis* spp. in sheep in China and has implications for the control of sheep sarcocystosis.

## 1. Introduction

Members of the genus *Sarcocystis* are protozoans that are widely parasitic in reptiles, birds, rodents, livestock, fish, and occasionally in humans [1]. *Sarcocystis* spp. require two hosts to complete their life cycle, usually herbivores as intermediate hosts and carnivores as definitive hosts. Sarcocysts form in the tissues of intermediate hosts, whereas sporocysts develop in the small intestine of the definitive host [2]. Sarcocystosis can result in fever, lethargy, stunted growth, poor appetite, lameness, and abortion, in severe cases, lead to death [3]. Intermediate hosts acquire infection by ingesting food or water that are contaminated with *Sarcocystis* oocysts or sporocysts. Humans can be either definitive hosts or intermediate hosts [4]. Humans as definitive hosts can be infected with the *Sarcocystis* species by ingestion of contaminated food or water and can have symptoms such as abdominal pain, diarrhea, anorexia, vomiting, and in severe cases, anemia and necrotizing enterocolitis can occur [5,6]. Sarcocysts in humans have been found in skeletal muscle and cardiac muscle, and *Sarcocystis* spp. are considered one of the most important parasitic protozoans threatening human health globally [5,6].

Currently, more than 200 species of *Sarcocystis* have been recorded in different animals [7]. At least six species of *Sarcocystis* have been described in sheep (*Ovis aries*) as intermediate hosts, namely, *S. arieticanis*, *S. tenella*, *S. gigantea*, *S. medusiformis*, *S. mihoensis*, and *S. microps* [8]. Among them, *S. arieticanis* is slightly less pathogenic than *S. tenella* [9,10]. Sheep can be infected with these protozoans at any time under natural conditions [11]. The infection of *Sarcocystis* spp. in domestic sheep occurs worldwide, with prevalence ranging from 9 to 100% [5], which can lead to problems such as degradation of meat product quality, jeopardization of public health, and causation of economic losses to the livestock industry. Therefore, the prevention and control of *Sarcocystis* infection in humans and animals is very important [12].

Sheep and goats (*Capra hircus*) are the first livestock to be domesticated by humans [13], yielding a diverse range of invaluable products. They can provide meat, high-protein milk, and high-quality cashmere products for human beings [14]. Lamb meat can provide abundant protein, fatty acids, vitamins, and minerals to humans [15], thus sheep farming is considered an important sector of agriculture in China. As people’s quality of life improves, domestic demand for sheep products has also increased significantly [16].

Up to now, most molecular studies on *Sarcocystis* spp. have been based on the amplification and sequencing of the 18S ribosomal RNA (rRNA) gene, 28S rRNA gene, and mitochondrial cytochrome c oxidase subunit I (*cox*1) gene. According to several studies, it has been found that the 18S rRNA gene and 28S rRNA genes are not quite suitable for distinguishing closely related species, and its identification accuracy is inferior to that of the c*ox*1 gene [17,18]. The *cox*1 gene is considered to be an ideal genetic marker in taxonomic identification and population genetics of *Sarcocystis* spp. [19,20]. In China, the annual production of sheep meat in 2017 was 4851 thousand tons [21]. However, surveys of *Sarcocystis* prevalence have been conducted only in a few Chinese provinces in the past [22], and there are no data on *Sarcocystis* spp. infection in sheep in Shanxi Province, which is an important province in sheep farming. Thus, the objectives of the present study were to examine whether sheep in Shanxi Province were infected with *Sarcocystis* spp. and to reveal the prevalence and geographical distribution of *Sarcocystis* spp. in sheep in Shanxi Province by PCR amplification and sequencing analyses of the mitochondrial *cox*1 gene, which will provide base-line data for executing intervention measures against *Sarcocystis* spp. infection in sheep in this province.

## 2. Materials and Methods

### 2.1. Sample Collection

From October to November 2023, fresh muscle tissue samples (3–5 g) of sheep were purchased from farmers’ markets from each animal. In total, 582 muscle samples (inner ridge, neck meat, front leg, and rear leg) of sheep were collected, among which 226, 124, and 232 samples were collected from Northern Shanxi Province (Youyu, Huairen, and Hunyuan), Central Shanxi Province (Qi, Taigu, and Pingyao), and Southern Shanxi Province (Fushan, Jishan, Xia, and Hejin), respectively (Table 1). Each sample was individually collected into a sample bag and labeled with the relevant details, such as types of tissues and geographic location, then kept in a foam box at a low temperature. After collection, the tissue samples were transported to the Laboratory of Parasitic Diseases at the College of Veterinary Medicine, Shanxi Agricultural University until further analysis and were frozen in a −20 °C freezer until the extraction of genomic DNA.

### 2.2. DNA Extraction and PCR Amplification

For each sheep tissue sample, approximately 200 mg of muscle fiber was used to extract the genomic DNA by using the TIANamp Genomic DNA Kit (Tiangen Biotech, Beijing, China) following the manufacturer’s specifications, and the DNA samples were stored in a freezer at −20 °C until further analysis.

The *cox*1 gene was amplified from each DNA sample by PCR using the primers SF1 [23] and SR9 [24], and the length of the amplified products was approximately 1100 bp. PCR reactions were carried out in volumes of 25 µL mixture containing 2.5 µL of 10 × PCR buffer (Mg^2+^ plus), 2 µL of DNA samples, 2 µL of dNTP, 1 µL of each primer, 0.5 µL of Ex *Taq* DNA polymerase (5 U/µL), and 16 µL of ddH_2_O. The PCR reaction conditions were as follows: initial denaturation (95 °C for 5 min); followed by 35 cycles denaturation (94 °C for 50 s), annealing (52 °C for 1 min), and extension at (72 °C for 1 min); and a final extension (72 °C for 10 min). To ensure the reliability of the results, the positive control (verified DNA of *Sarcocystis* spp. by sequencing), *Sarcocystis*-negative sheep muscle DNA and negative control (reagent-grade water) were added to each PCR assay. All PCR products were examined by gel electrophoresis using 1% agarose with ethidium bromide. The positive PCR products were sent to Sangon Biotech Ltd. (Shanghai, China) for bidirectional sequencing.

### 2.3. Sequence and Phylogenetic Analyses

In order to determine the *Sarcocystis* species, the obtained *cox*1 sequences were compared with those of *Sarcocystis* spp. deposited previously in GenBank using BLAST. Ambiguous sequences were excluded, since they may represent sequencing errors or mixed infections of *Sarcocystis* spp. Only those pure sequences with no double peaks or indels were further analyzed. The representative sequences of *Sarcocystis* spp. from sheep detected in this study were deposited in the GenBank with accession numbers PQ189447, PQ189448, and PQ165949. The phylogenetic tree was re-constructed using the neighbor-joining (NJ) method and Kimura two-parameter (K2P) model in the MEGA 7.0 software [22]. One thousand replicates (bootstrap value) were selected to determine the support for the clades generated. *Toxoplasma gondii* (GenBank accession No. JX473253) was used as the outgroup.

### 2.4. Histopathology

The positive mutton samples (verified by sequencing of *cox*1 gene) were fixed in 10% formalin buffer and embedded in paraffin wax. Sections (0.5 μm thick) were prepared. The tissue was fixed on glass slides for hematoxylin and eosin staining. The histological sections were scanned for *Sarcocystis,* and all the pathological changes were thoroughly evaluated with a light microscope (Olympus BX43, Olympus, Japan).

### 2.5. Statistical Analysis

In this study, the prevalence of *Sarcocystis* spp. infection among different regions was statistically analyzed by the chi-square test using SPSS V20.0 (IBM, Chicago, IL, USA). The difference is considered significant when the *p*-value is <0.05. Odds ratios (ORs) and their 95% confidence intervals (95% CIs) based on likelihood ratio statistics were also estimated to explore the strength of the association between prevalence and test conditions [25].

## 3. Results

### 3.1. Prevalence of Sarcocystis spp. in Sheep in Shanxi Province

**Figure 1:**
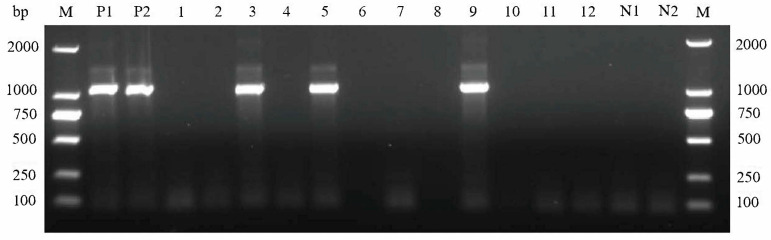
PCR products amplified from sheep muscle samples targeting the mitochondrial cytochrome c oxidase subunit I (*cox*1) gene of *Sarcocystis* spp, with template DNA from reference samples of *S. tenella* (P1) and *S. arieticanis* (P2). Lanes 1–12 represent the PCR results of DNA samples extracted from representative sheep muscle samples. Lane N1 represents negative control, N2 represents *Sarcocystis*-negative sheep as a control, and lane M represents DNA size markers.

In this study, based on the amplification of the *cox*1 gene, 197 out of 582 sheep tissue samples were detected as positive for *Sarcocystis*, with an overall prevalence of 33.85% (197/582) (Table 1). The prevalence of *Sarcocystis* spp. in sheep ranged from 19.67% to 58.97% in ten counties located in Shanxi Province. Among the 197 positive samples, 56 samples were from Northern Shanxi, 48 samples were from Central Shanxi, and 93 samples were from Southern Shanxi. *Sarcocystis* prevalence in sheep among the ten areas was significantly different (χ2 = 32.63, *p* < 0.001). The PCR results of some representative sheep muscle samples are shown in Figure 1, and the original gel image is available in the Supplementary Materials (Figure S1).

### 3.2. Sarcocysts in the Muscle of Sheep

**Figure 2:**
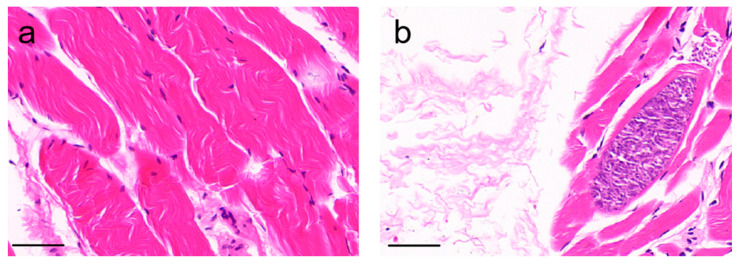
HE staining of *Sarcocystis* in sheep muscle tissue. (**a**) Sheep muscle tissue not infected by *Sarcocystis*. (**b**) The sarcocysts from *Sarcocystis* in the infected sheep muscle tissue. Scale bar: 50 µm.

The cysts were blue-purple in color and were parallel to the muscles, which were pink in color. The cyst wall was smooth and thin, and the cyst cavity was full of crescent-shaped bradyzoites (Figure 2).

### 3.3. Phylogenetic Analysis

The obtained 197 *cox*1 sequences were aligned and compared with the corresponding *cox*1 gene sequences of *Sarcocystis* spp. available in the GenBank. Among them, 196 *cox*1 sequences shared the highest nucleotide similarities with those of *S. tenella*, ranging from 98.56% to 99.81%, followed by *S. capracanis* (OP470343 and OP470344) from *Capra hircus* (93.36–93.46%, on average 93.41% identity), and *S. heydorni* (KX057995 and KX057994) from *Bos taurus* (90.10–90.29% identity, on average 90.20% identity). The remaining one *cox*1 sequence showed the highest nucleotide similarity (99.71%) with that of *S. arieticanis*, followed by *S. hircicanis* (KU820975 and KU820976) from *Capra hircus* (92.28–92.57%, on average 92.43% identity), and S. *grueneri* (KC209615–KC209623) from *Rangifer tarandus* (83.83–84.47% identity, on average 84.15% identity). The obtained 197 *cox*1 sequences of *S. tenella* in this study showed nucleotide similarities ranging from 98.12% to 100.00%. Notably, *S. tenella* were detected in sheep in all cities, whereas *S. arieticanis* was identified only in one county, namely, Hejin County, suggesting that the infection of sheep in Shanxi Province was mainly dominated by *S. tenella*.

**Figure 3:**
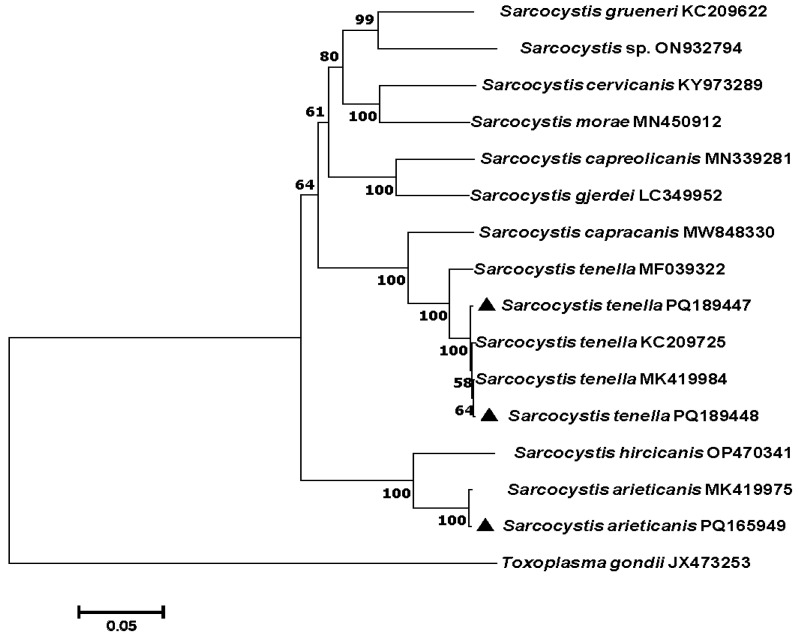
Phylogenetic relationships of *Sarcocystis* spp. based on *cox*1 gene sequences using the neighbor-joining (NJ) method. *Toxoplasma gondii* was used as the outgroup. Representative *Sarcocystis cox*1 sequences obtained by this study are labeled with black triangle (▲). The bootstrap value is shown when >50%.

For phylogenetic analysis of *Sarcocystis* spp., two representative *cox*1 sequences of *S. tenella* (accession nos. PQ189447 and PQ189448) and one *cox*1 sequence of *S. arieticanis* (accession no. PQ165949) obtained from the present study were used to re-construct the phylogenetic tree with other relevant *Sarcocystis* spp. In the phylogenetic tree inferred from *cox*1 sequences (Figure 3), the newly sequenced *S. tenella* isolates (PQ189447 and PQ189448) clustered with *S. tenella* (KC209725) from *Ovis aries* and *S. tenalla* (MK419984) from *Ovis aries*, which then formed a clade with *S. capracanis* (MF039322) from goat. The newly sequenced *S. arieticanis* isolate (PQ165949) clustered with *S. arieticanis* (MK419975) from *Ovis aries*, which then formed a clade with *S. hircaicanis* (OP470341) from goat.

## 4. Discussion

Sarcocystosis is a zoonotic parasitic disease. *Sarcocystis* spp. infections can lead to a decline in the quality of livestock products, causing economic losses to the livestock industry and public health problems. *Sarcocystis* spp. in sheep have been reported both domestically and internationally and are widely distributed in Oceania (93.31%, 990/1061) [5], Europe (23.28%, 179/769) [26], Africa (54.67%, 621/1136) [5], South America (95.83%, 115/120) [27], North America (67.40%, 1141/1693) [5], and Asia (48.02%, 26061/54270) [28]. In the present study, the overall *Sarcocystis* spp. prevalence in sheep in Shanxi Province was 33.85% (197/582), which was lower than that in Iran (63.83%) [3], Brazil (95.80%) [27], Mongolia (96.90%) [28], and some provinces of China, such as Qinghai Province (49.96%) [22]. However, the *Sarcocystis* spp. prevalence in sheep in Shanxi Province was higher than that in Gansu Province (7.74%), Henan Province (11.60%), and Liaoning Province (27.83%) [29].

In this study, the highest *Sarcocystis* spp. prevalence in sheep was detected in Qi County (58.97%, 23/39), followed by Fushan County (54.39%, 31/57) and Jishan County (37.93%, 33/87) in Shanxi Province. Qi County is in close proximity to Taiyuan city, the capital of Shanxi Province, which has a huge population and convenient transportation, which may facilitate the spread of pathogens, and these may have contributed to the highest rate of *Sarcocystis* infection in sheep in Qi County. We found that the prevalence of *Sarcocystis* spp. in sheep gradually decreased with increasing latitude. This is probably because mild and warm climate could be conducive to the survival and dissemination of *Sarcocystis*. spp. Fushan County and Jishan County is closer to the equator than other cities, which may contribute to their relatively high rates of *Sarcocystis* spp. infection in sheep.

Notably, only two *Sarcocystis* species, namely, *S. tenella* and S. *arieticanis*, were identified in sheep in Shanxi Province in the present study. Both *Sarcocystis* species form microcysts in muscle tissues and are transmitted by canids, i.e., sporulated oocysts shed in the feces of infected canids [5]. In Shanxi Province, domestic dogs are raised frequently to safeguard the sheepfold, which greatly increases opportunities for the livestock to contact the feces of domestic dogs; this could facilitate the efficient circulation of *S. tenella* and *S. arieticanis* between domesticated livestock (intermediate) hosts and domestic canines (definitive) hosts. Thus, we speculate that this is the primary factor for the popularity of *S. tenella* and *S. arieticanis* in sheep in Shanxi Province.

Traditional morphological methods for the identification of *Sarcocystis* species have limitations, such as their inability to distinguish between species with similar morphology and structure, and the process is also time-consuming and labor-intensive, whereas molecular approaches are able to rapidly and accurately differentiate *Sarcocystis* species compared to morphological methods [30]. The mitochondrial genome has a small molecular mass and a relatively stable structure. The *cox*1 gene is relatively conserved in the mitochondrial genome within a species and has been widely used for *Sarcocystis* species identification and genetic evolutionary studies in recent years [31,32]. The PCR method using *cox*1 as the target gene has excellent specificity and is suitable for the identification of *Sarcocystis* spp. in animals [33].

The prevention and control of *Sarcocystis* spp. in sheep is of importance. *Sarcocystis* spp. in sheep can cause economic losses to the livestock industry, reduce meat quality, and threaten public health [12]. Therefore, the relevant units should strengthen the feeding management of sheep, ensure forage and drinking water are clean, disinfect farms on a regular basis, strengthen the management of stray dogs and cats, and reduce the contact between these stray animals and sheep, as well as strengthen the relevant quarantine and pollution control [34].

## 5. Conclusions

The present study revealed, for the first time, an overall 33.85% prevalence of *Sarcocystis* spp. infection in sheep in Shanxi Province based on the amplification and sequencing of the mitochondrial *cox*1 gene. Two *Sarcocystis* species were identified, namely, *S. tenella* and *S. arieticanis*, with *S. tenella* being the predominant species. These results extend the geographical distribution and provide base-line data for executing intervention measures against *Sarcocystis* spp. in sheep in Shanxi Province and elsewhere.

## Figures and Tables

**Table 1:** Prevalence of *Sarcocystis* spp. in sheep in Shanxi Province, China.

Geographical Location	Category (County)	No. Tested	No. Positive	Prevalence % (95% CI)	Odds Ratio (95% CI)	*p*-Value
Northern Shanxi	Youyu	80	21	26.25 (16.61–35.89)	1.45 (0.65–3.25)	<0.001
	Huairen	85	23	27.06 (17.61–36.50)	1.52 (0.69–3.35)	
	Hunyuan	61	12	19.67 (9.70–29.65)	1	
Central Shanxi	Qi	39	23	58.97 (43.54–74.41)	5.87 (2.39–14.40)	
	Pingyao	50	15	30.00 (17.30–42.70)	1.75 (0.73–4.20)	
	Taigu	35	10	28.57 (13.61–43.54)	1.63 (0.62–4.30)	
Southern Shanxi	Jishan	87	33	37.93 (27.74–48.13)	2.50 (1.16–5.37)	
	Fushan	57	31	54.39(41.46–67.32)	4.87 (2.15–11.04)	
	Hejin	55	19	34.55 (21.98–47.11)	2.16 (0.93–5.00)	
	Xia	33	10	30.30 (14.62–45.98)	1.78 (0.67–4.71)	
Total		582	197	33.85 (30.00–37.69)		

## Data Availability

The data sets supporting the results of this article have been submitted to GenBank, and the accession numbers are shown in the article.

## Supplementary Materials

The following supporting information can be downloaded at: https://www.mdpi.com/article/10.3390/vetsci11100504/s1, Figure S1: Original Gel Image.
