# Supplementary figures and images for: First Molecular Identification and Prevalence of Sarcocystis spp. in Sheep Intended for Human Consumption in Shanxi Province, China
Source: Vet Sci. 2024 Oct 14;11(10):504. doi: 10.3390/vetsci11100504 (PMC11512325; doi:10.3390/vetsci11100504)

Supplementary materials

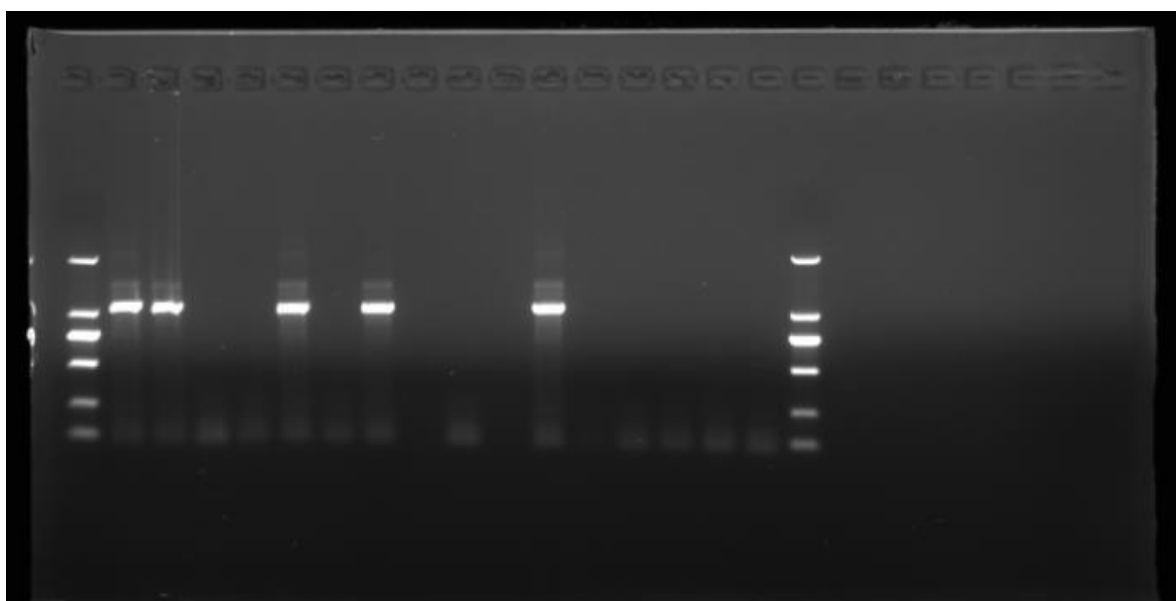

Figure S1: Original Gel Image

Supplement: Supplementary file 1 [file vetsci-11-00504-s001.zip › vetsci-3186188-supplementary.pdf]
